# Isolation and Culture of Larval Cells from *C. elegans*

**DOI:** 10.1371/journal.pone.0019505

**Published:** 2011-04-29

**Authors:** Sihui Zhang, Diya Banerjee, Jeffrey R. Kuhn

**Affiliations:** Department of Biological Sciences, Virginia Tech, Blacksburg, Virginia, United States of America; University of Texas MD Anderson Cancer Center, United States of America

## Abstract

Cell culture is an essential tool to study cell function. In *C. elegans* the ability to isolate and culture cells has been limited to embryonically derived cells. However, cells or blastomeres isolated from mixed stage embryos terminally differentiate within 24 hours of culture, thus precluding post-embryonic stage cell culture. We have developed an efficient and technically simple method for large-scale isolation and primary culture of larval-stage cells. We have optimized the treatment to maximize cell number and minimize cell death for each of the four larval stages. We obtained up to 7.8×10^4^ cells per microliter of packed larvae, and up to 97% of adherent cells isolated by this method were viable for at least 16 hours. Cultured larval cells showed stage-specific increases in both cell size and multinuclearity and expressed lineage- and cell type-specific reporters. The majority (81%) of larval cells isolated by our method were muscle cells that exhibited stage-specific phenotypes. L1 muscle cells developed 1 to 2 wide cytoplasmic processes, while L4 muscle cells developed 4 to 14 processes of various thicknesses. L4 muscle cells developed bands of myosin heavy chain A thick filaments at the cell center and spontaneously contracted *ex vivo*. Neurons constituted less than 10% of the isolated cells and the majority of neurons developed one or more long, microtubule-rich protrusions that terminated in actin-rich growth cones. In addition to cells such as muscle and neuron that are high abundance *in vivo*, we were also able to isolate M-lineage cells that constitute less than 0.2% of cells *in vivo*. Our novel method of cell isolation extends *C. elegans* cell culture to larval developmental stages, and allows use of the wealth of cell culture tools, such as cell sorting, electrophysiology, co-culture, and high-resolution imaging of subcellular dynamics, in investigation of post-embryonic development and physiology.

## Introduction

*Caenorhabditis elegans* is used widely as a genetic and developmental model organism because of its simple anatomy, invariant cell lineage, compact genome, and the wealth of genetic tools available for its study. However, high-throughput access to individual cells has been limited to embryonic lineages. Early work showed that embryonic cells from dissociated blastomeres could be cultured for short periods of time and were capable of partial differentiation *in vitro* [1], [2], [3]. Building upon this early work, Bloom systematically tested a variety of cell isolation techniques and conditions for larger scale embryonic cell culture [4]. Bloom's work was expanded and optimized upon by Strange and colleagues who introduced a method of embryonic cell culture to the wider *C. elegans* community [5], [6], [7]. Large-scale embryonic cell culture expanded the available experimental repertoire to include electrophysiological analysis of cultured neurons and muscles [5], [8], isolation of specific cell types by automated cell sorting [9], [10], cell type specific gene expression profiling [7], [11], [12], assessing the effect of environmental toxins on cultured cells [13], [14], dissecting cellular mechanisms of RNA interference [15], [16], and high-resolution total internal reflection fluorescence microscopy of subcellular events [17].

Although embryonic cell culture has allowed new advances in cell and tissue-specific studies in *C. elegans*, it is not without limitations. Embryonically derived cells differentiate within 24 hours to resemble L1 stage cells [5], [6]. Development of key tissue and organ systems, such as the reproductive system and neuro-epithelial tissues [18], occurs after hatching, and many cells do not gain their full functionalities until later larval stages [19]. These post-embryonic developmental events, and the molecular mechanisms that control them, cannot be studied using cultured embryonic cells.

The ability to access and manipulate larval stage cells would greatly benefit cell and tissue specific studies of post-embryonic developmental events. However, there are no current reports of successful isolation of larval stage cells, and former attempts appear to have been hindered by the tough and relatively impermeable cuticle that encapsulates the worm and prevents access to cells and tissues [20]. An alternative approach to gaining access to larval cells and organs is dissection of individual animals [21], [22], [23], [24]. However, dissection is both technically-demanding and can only be performed on a small scale. Tagging of mRNA is a molecular approach to cell-specific studies that can be carried out in whole worms without isolating cells, and has been used to profile gene expression in specific larval cell types [25], [26]. However, mRNA tagging suffers from several experimental constraints, such as the need for cell-specific promoters, and is limited to providing transcriptional information [26], [27]. To circumvent the limitations of using dissection and mRNA tagging to access *C. elegans* post-embryonic cells, we have developed a technically simple method for large-scale isolation of cells from *C. elegans* larvae. Large quantities of viable larval cells from synchronized L1 to L4 stage worms can be isolated using this method and used for cell and tissue specific studies of post-embryonic cellular phenomena.

## Results

### Effective disruption of the larval cuticle to release cells

**Figure 1:**
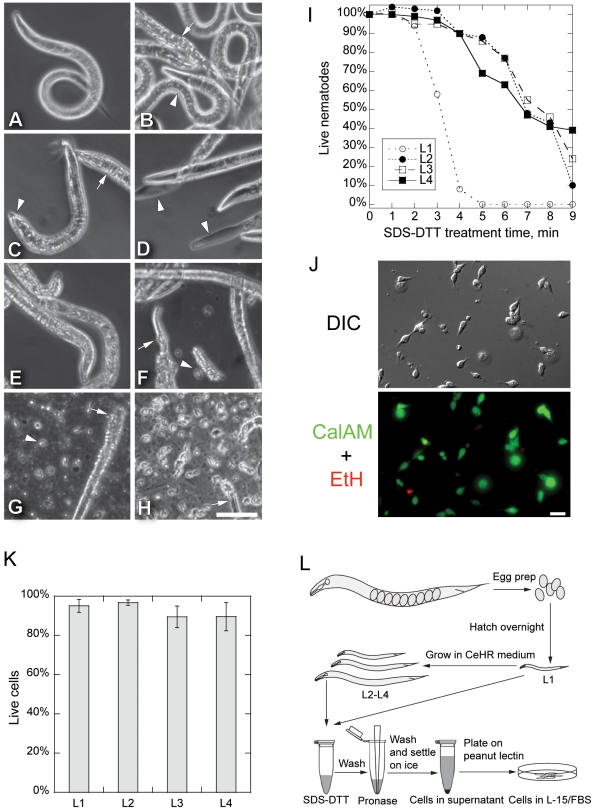
Larval Cell Isolation Procedure. (**A–H**) Phase contrast micrographs of L3 nematodes during cell isolation. (**A**) Untreated L3 worms had smooth body outlines and normal bending motion. (**B**) Nematodes treated with 15 mg/ml pronase for 20 min did not dissociate during repeated pipetting. Most nematodes remained intact (*arrowhead*), but some developed a rougher cuticle (*arrow*). (**C**) Nematodes treated with SDS-DTT for 4 min remained intact. Treated nematodes swelled slightly, especially at the head (*arrowhead*), and cuticle wrinkles appeared (*arrow*). However, nematodes continued to move. (**D**) Longer SDS-DTT treatment (8 min) killed nematodes. Some showed cuticle regions devoid of cells (*arrowheads*). (**E**) A short 4 min SDS-DTT treatment followed by repeated pipetting in egg buffer alone did not dissociate nematodes. (**F**) Adding pronase after a short SDS-DTT treatment (4 min) began to digest the cuticle. By 10 min, some nematodes lost cuticle integrity and released cells (*arrowhead*). Arrow points to an exposed pharynx in a partially digested worm. (**G**) Repeated pipetting during pronase digestion of SDS-DTT treated (4 min) nematodes over 20 min completely digested most worms. More cells were released (*arrowhead*) with pipetting than without. Some partially digested worms remained (*arrow*). (**H**) After digested worms were settled for 30 min on ice, the supernatant mostly contained cells and little nematode debris (*arrow*). Large worm debris but few cells settled into the pellet (not shown). Scale bar in A–H is 50 µm. (**I**) L2–L4 stage nematodes survived longer in SDS-DTT than did L1 nematodes. Nematodes were scored as dead if they were rigid without any bending activity or had dissolved leaving empty cuticles. Live/dead scores were normalized to worms incubated for 10 min in egg buffer (0 min). (**J**) DIC and fluorescence micrographs of live/dead cell assay of adherent larval cells one day after plating. Calcein-AM stains for live cells (green), and ethidium homodimer indicates dead cells (red). Scale bar: 10 µm. (**K**) Viability of adherent L1–L4 larval cells tested by Calcein-AM and ethidium homodimer staining one day after isolation. Error bar is SD of three observations (L1: n = 819; L2: n = 417; L3: n = 485; L4: n = 741). (**L**) Schematic diagram of larval cell isolation procedure. Eggs are isolated from gravid adults and hatched overnight. L1 larval cells are isolated immediately, while larvae are grown in CeHR medium for L2 to L4 stage cell isolation. Nematodes are treated with SDS and DTT for 2–4 min, washed with egg buffer, and incubated with pronase for 8–20 min with gentle pipetting. Cells were separated from debris by settling on ice for 30 min, plated onto penut lectin coated glass substrates and maintained in L-15/fetal bovine serum medium.

The cuticle is the primary barrier to accessing cells and tissues in *C. elegans* larvae and adults. Larval and adult cuticles are composed primarily of collagens, highly cross-linked cuticlins, and surface glycoproteins [28]. We tested the ability of a range of proteases, including elastase, pepsin, α-chymotrypsin, pronase, and a cocktail of collagenases to dissolve the cuticle and release cells. However, none of these reagents affected the integrity of the larval cuticle (not shown). We therefore sought a method that would break down the extensive disulfide bonds and di- and tri-tyrosine crosslinks that strengthen the cuticle, and would thus make the cuticle more accessible to protease digestion. The anionic detergent sodium dodecyl sulfate (SDS) and reducing agents denature proteins and are known to weaken the cuticle. In studies where the aim was to solubilize and extract the cuticle, Cox and colleagues [29] showed that treatment with SDS and a 5% solution of the reducing agent ß-mercaptoethanol, along with sonication and heating, solubilized 69% of cuticle content. Austin and colleagues [30] employed a similar method, but with shorter and gentler treatment, using 0.25% SDS to dissolve the cuticle while preserving epithelial seam cell contacts. We found that incubation of nematodes in 0.25% SDS and 3% of the reducing agent dithiothreitol (DTT) for 2 to 4 minutes at room temperature altered the appearance of the cuticle without disrupting nematode body integrity (Figure **1**). The anterior portion of the heads of SDS-DTT treated animals protruded rather than being smoothly linked to the more posterior part, and the cuticle wrinkled, indicating the disruption and loosening of cuticular structure (Figure **1**C). Nematodes incubated in SDS-DTT swelled to resemble a “dumpy” phenotype and showed reduced mobility, traits that are associated with mutations that decrease cuticle integrity [31]. When animals were treated for longer times, the majority of worms became stiff and immobile and lacked wild type movement. Extensive SDS-DTT treatment eventually solubilized worms completely, leaving empty cuticle husks (Figure **1**D). Although animals treated with SDS-DTT for short times were less active and lost sinusoidal movement, they still twitched, indicating nervous system and musculature function.

To evaluate the effect of SDS-DTT treatment on animal survival, we determined survival rates over 9 minutes of SDS-DTT treatment (Figure **1**I). We found that 94% of L1 worms survived after 2 minutes of SDS-DTT treatment, but that survival dropped rapidly to 58% at 3 minutes of exposure. In contrast, SDS-DTT treatment killed L2 through L4 worms more slowly. At 4 minutes, 90% of L2 to L4 worms remained alive and survival did not decrease below 50% until 7 to 8 minutes. The decreasing sensitivities to SDS-DTT treatment for L1 through L4 worms are consistent with changes in cuticle composition and structure over larval development. For example, L4 larvae are likely more resistant to SDS-DTT treatment because L4 cuticles are approximately 2.5 times thicker than L1 cuticles [32].

Having established a treatment that weakens the cuticle without causing extensive death, we sought to identify compounds that could disrupt the cuticle and release live cells from larval worms. Mechanical treatments of SDS-DTT treated worms, including repeated pipetting, were ineffective in releasing cells (Figure **1**E). Treatment with either of the proteinases pepsin or α-chymotrypsin also did not release cells from SDS-DTT treated worms. However, it was previously demonstrated that the proteinase elastase can digest both basal and cortical cuticle layers of SDS-purified cuticles, and that the proteinase pronase can digest the basal cuticle layer and the pharyngeal cuticle [29]. We thus tested these two proteinases and found that while elastase digested only 10% of SDS-DTT treated worm, addition of 15 mg/ml pronase to SDS-DTT treated worms (Figure **1**F) resulted in digestion of 70% (L1) to 96% (L4) of cuticles. When combined with mechanical disruption by pipetting, pronase and SDS-DTT treatment dissociated tissues and released single cells very efficiently (Figure **1**G–H). However, L2 to L4 nematodes required 2.5- to 3-fold longer incubation time in pronase compared to L1 nematodes for efficient digestion, reflecting the increased thickness of older larval cuticles. For both L1 and older larvae, pronase treatment alone was ineffective in digesting cuticles and pre-sensitization by SDS-DTT treatment was required for efficient pronase-mediated cuticular digestion (Figure **1**B).

### Primary culture of isolated larval cells

#### Cell yields

SDS-DTT-pronase treatment and mechanical disruption of L1 worms yielded 2.7±1.9×10^4^ (mean ± S.D., n = 3 independent isolations) cells in solution per microliter (µl) of packed nematodes. With approximately 1.1×10^4^ packed L1 animals per µl (n = 1) and typical yields of approximately 40 µl of packed worms from three 100 mm diameter feeding plates seeded with a full lawn of bacteria, we obtained 1.1±0.8×10^6^ cells during a typical L1 isolation, or approximately 2.5±1.7 cells per animal. Cell isolation from L2 to L4 larvae was more efficient, yielding 7.8±1.7×10^4^ cells per µl of packed nematodes (n = 4).

An accurate comparison of the efficiencies of larval and embryonic cell isolation is difficult because of the large ranges in cell number and cell size among embryonic and different larval stages, and variability in the numbers of packed embryos or larvae in a given volume. Nevertheless, we provide a rough comparison of cell yields based on equivalent volumes of packed embryos and larvae. We used the method of Christensen et al [5], [6] to isolate embryonic cells and obtained 6.1±2.9×10^4^ pre-adhered cells per µl of packed eggs (n = 3). At a density of 1.6×10^4^ packed eggs per µl (n = 1), embryonic cell isolation produced 3.8±1.8 cells per egg. Approximately 40 µl of packed eggs yielded 2.4±1.2×10^6^ pre-adherent cells. Thus, our method of larval cell isolation (2.7±1.9×10^4^ to 7.8±1.7×10^4^ pre-adherent cells per µl of packed larvae) provides cell yields in the same order of magnitude as embryonic cells (6.1±2.9×10^4^ pre-adherent cells per µl of packed eggs) isolated by the method of Christensen et al [5], [6]. Both embryonic and larval cell isolation protocols provide cell yields that are sufficient for methods, such as cell sorting, that require large quantities of cells [9], [10].

#### Adherence to substrate

We tested a number of molecules, including laminin, poly-D-lysine, fibronectin, and collagen IV, which are commonly used in cell culture to enable adherence of cells to a substrate. The larval cells showed maximum adherence to glass surfaces plated with 0.5 mg/ml peanut lectin, which was subsequently used for all isolations. Cells adhered less well to poly-D-lysine and fibronectin, and did not adhere at all to laminin or collagen IV coated surfaces. Isolated larval cells were typically plated at a density of 5 to 6×10^6^ cells/ml, and were maintained in commercially available L-15 culture medium supplemented with fetal bovine serum albumin at an osmolarity of 340 mOsm. The plating density and the culture conditions are similar to those optimized for maintenance of *C. elegans* embryonic cells [5], [6]. For example, cells were allowed to adhere overnight and non-adhered cells were washed away. A typical density of adherent cells isolated from L1 stage worms was 3.8±1.3×10^3^ cells/mm^2^ (n = 2) spread over a total area of 200 mm^2^, yielding 7.8±2.4×10^5^ total adherent cells, or approximately 70% of the cells in solution before plating.

#### Survival in culture

The SDS-DTT-pronase treatment and culture conditions described did not significantly damage adherent larval cells. We simultaneously monitored live and dead cells using a two-color fluorescence assay with Calcein acetoxymethyl ester (Calcein-AM), which measures the population of live cells, and ethidium homodimer, which measures the population of dead cells [33]. These cytotoxicity/cytoviability assays showed that 80% to 97% of adherent larval cells were viable after 16 hrs in culture (Figure **1**K).

#### Bacterial contamination

In our initial attempts at isolating and culturing larval cells, we grew *C. elegans* larval populations on bacterial lawns plated on solid media. While we obtained substantial yields of viable larval cells, the cell culture would frequently become contaminated with bacterial populations that overwhelmed the antibiotics in the culture medium. However, cells isolated from L1 larvae that were hatched and grown only in sterile M9 medium remained uncontaminated, indicating that the contaminating bacteria in older larval cell cultures were likely from the ‘food’ lawns and had survived the SDS-DTT-pronase treatment. To circumvent bacterial contamination, we grew synchronized larval populations under axenic conditions in sterile CeHR medium [34], [35], which successfully prevented bacterial growth in the cultured cells (Figure **1**L).

### Cells can be isolated from both high and low abundance larval cell types

**Figure 2:**
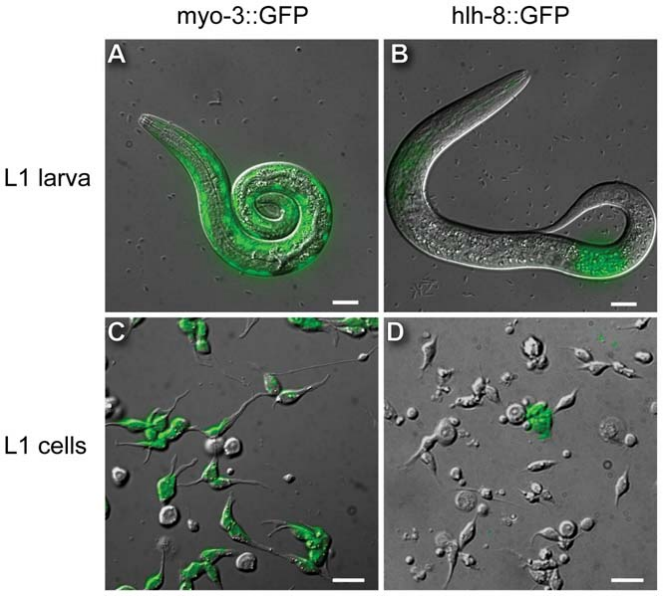
Fluorescent and DIC merge micrographs of muscle- or M-lineage-specific GFP expression in L1 larvae and L1 cell isolates. (A) L1 worm of strain PD4251/*myo-3*::GFP, which has nuclear and mitochondrial GFP expression (green) in body wall muscle cells. (B) L1 worm of strain NH3402/*hlh-8*::GFP, which expresses membrane-bound GFP (green) in the M cell (posterior) and a small number of neuron-like cells in the head (anterior). Both animal heads are positioned to the left. (C) One-day culture of cells from L1 PD4251/*myo-3*::GFP. Green shows GFP expression in mostly bipolar, spindle-shaped muscle cells. (D) One-day culture of cells from L1 NH3402/*hlh-8*::GFP. Green shows GFP expression in a squamous-shaped M cell. All scale bars are 10 µm.

We used GFP reporter strains to identify some of the cell types isolated from larval worms using the SDS-DTT-pronase method. We observed expression of *myo-3*::GFP, which represents expression of a body wall muscle cell specific myosin [36], [37], in approximately 81% of L1 derived cultured cells (Figure **2**, Table 1). We also observed expression of *unc-119*::GFP, which is expressed primarily in neural cells and a small number of muscle cells [38], in less than 10% of L1 derived cultured cells. *In vivo*, muscles constitute 15% (81/558) and neurons constitute 40% (222/558) of all cells in L1 larvae. Thus, our cell isolation method appears to enrich for muscle cells but not neural cells.

**Table 1:** *In vivo* and *in vitro* frequencies of muscle cells and M lineage cells in L1 larvae.

Strain	Marker	L1 Expression pattern	Frequency of cell type	
			*in vivo* 1	*in vitro* 2
PD4251	*myo-3::NGFP-lacZ; myo-3::MtGFP*	body wall muscles	15%	81±5% (1128, 3)
NH3402	*hlh-8::GFP-CAAX*	M lineage cells and a subset of head neurons	0.18%	1.0±0.1% (1152, 2)

1 *In vivo* frequencies were calculated from Sulston and Horvitz [19] based on cell numbers at hatching.

2 *In vitro* frequencies were measured by counting cells in 5 to 8 random fields of cells plated on peanut lectin one day after isolation. Values given as mean ± error (cells counted, no. of trials).

We tested whether our cell isolation method could extract larval cells that are present *in vivo* at a substantially lower density than muscle and neural cells types. The *hlh-8::*GFP reporter is expressed in the M lineage mesodermal cells, which constitute only 0.18% (1/558) of the total cell population of L1 larvae [39] (Figure **2**). We found that GFP positive cells constituted approximately 1% of the cells isolated and cultured from *hlh-8*::GFP larval populations, indicating the presence and enrichment of M lineage cells in larval cell culture (Figure **2**). Therefore, our SDS-DTT-pronase method is capable of isolating both high and low abundance cell types in larval *C. elegans*. However, it is unlikely that every larval cell type can be successfully isolated and our protocol will need to be tested and optimized for isolation of specific cell lineages.

### Isolated larval cells express cell type-specific morphologies and exhibit cellular activities *in vitro*

**Figure 3:**
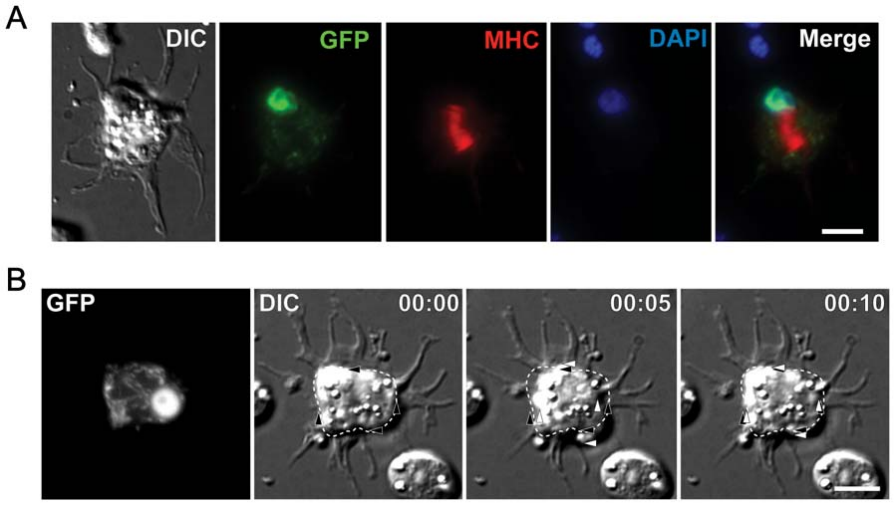
Muscle cells from L4 larvae express muscle-specific myosin and spontaneously contract *in vitro*. (**A**) DIC and fluorescence micrographs of fixed muscle cells isolated from L4 stage *myo3*::GFP worms, which expresses GFP in both nucleus and mitochondria of muscle cells. Cells were immunostained for myosin heavy chain A (MHC-A), and stained for nuclei (DAPI). A wide band of myosin was observed near the cell center. (**B**) Time-lapse DIC series of spontaneous contraction in a muscle cell from *myo3*::GFP reporter strain. GFP indicates muscle identity. Dotted outlines show cell body shape as at 0 second, and are static throughout the sequence. Arrowheads show edges of cell body. Black arrowheads indicate initial boundary and are static throughout the sequence. White arrowheads indicate the newest cell edge positions. The overall cell shape changes from near rectangular (0 sec) to oval (5 sec) and back to near rectangular (10 sec). Time is in min∶sec. Scale bars are 5 µm.

To evaluate whether isolated larval cells maintain cell type specific characteristics *in vitro*, we examined the GFP-positive cells from *unc-119*::GFP and *myo-3*::GFP strains for neural and muscle cell associated proteins and structures. Muscle cells are characterized by the presence of sarcomeres, which are contractile filament bundles that consist primarily of actin filaments and the motor protein myosin II. Myosins act by contracting actin filaments and can generate changes in cell shape. *C. elegans* larvae express two isoforms of myosin II heavy chain (MHC) in body wall muscles [40], [41], [42], [43]. MYO-3/MHC-A is found in the center of the A band of thick filaments, while the more abundant UNC-54/MHC-B isoform is found throughout the distal tips of the A band [44], [45]. We used a *myo-3*::GFP reporter strain to locate body wall muscle cells *in vitro* (Figure **3**A). Antibody staining of MHC-A in isolated *myo-3*::GFP larval muscle cells showed thin bands that were restricted to the center of the cell body, consistent with MYO-3/MHC-A localization to the center of the A band *in vivo*. These myosin structures in L4 isolated muscle cells functioned as sarcomeres as seven of the eight *myo-3*::GFP expressing L4 muscle cells showed spontaneous and repeated contractions over 30 minutes (Figure **3**B). Observations of five *myo-3*::GFP expressing cells for 30 minutes each showed an average of 0.9±0.3 contractions/min (mean ± SD, n = 5), with each contraction lasting 11.5±1.6 seconds, and intervals of 102±63 seconds of relaxation between contractions.

**Figure 4:**
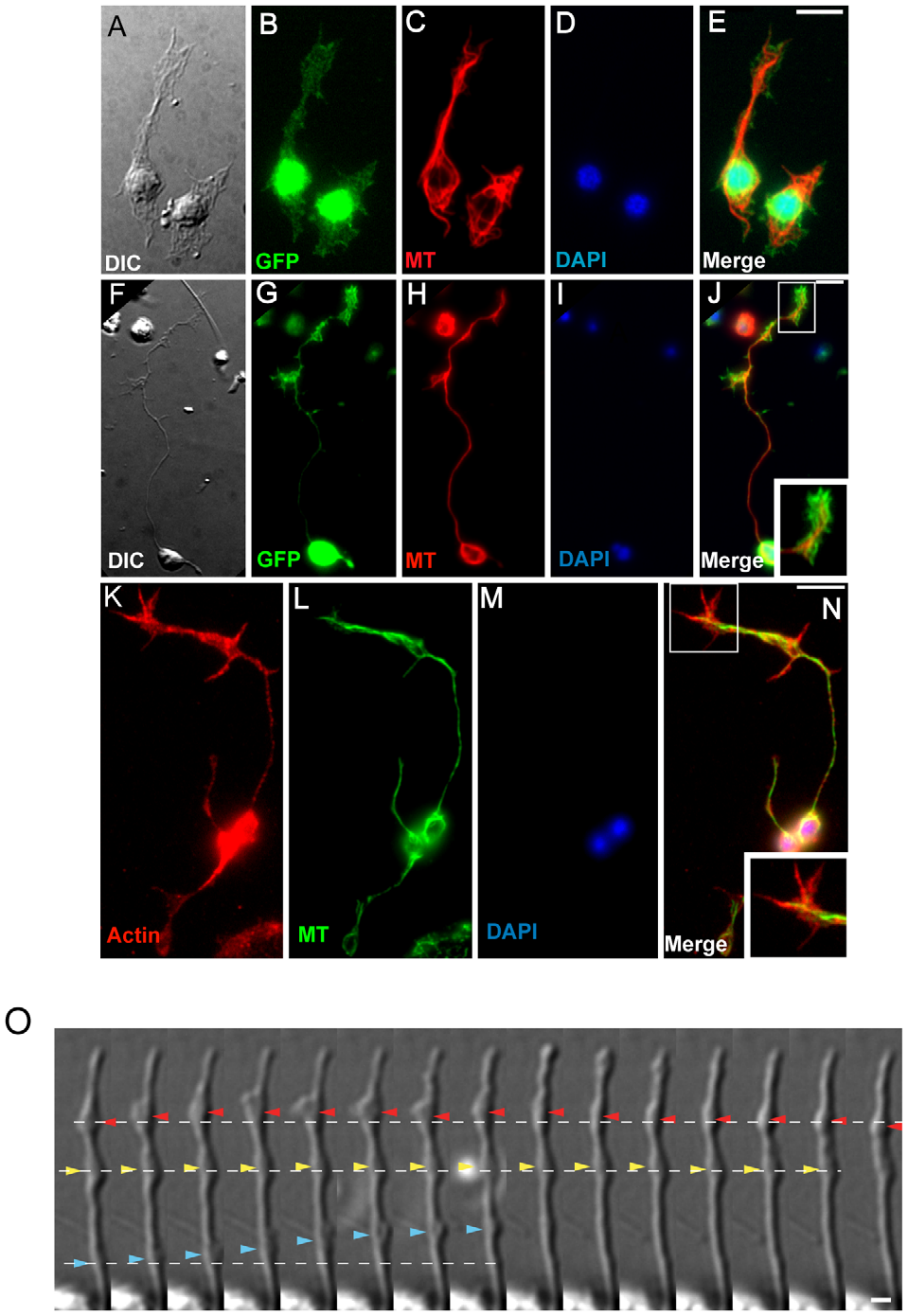
Cultured larval neurons extend projections and growth cones *in vitro*. (**A–E**) Microtubule network and cytoplasmic projections in GFP expressing cells isolated at L1 stage from an *unc-119*::GFP neuronal maker line. (**A**) DIC image of *unc-119*::GFP positive cells, (**B**) GFP expression in cell bodies (green), (**C**) anti-tubulin staining showing microtubules (red), (**D**) DAPI staining showing nuclei (blue), and (**E**) merged fluorescent images of fixed cells. (**F–J**) Microtubule network and cellular extensions in GFP expressing neurons after two days in culture. Staining as in A–E. (**J**) Enlarged inset shows a growth cone. (**K–N**) Actin and microtubule networks in neurons isolated from L1 stage N2/wildtype nematodes and fixed after 1 day. (**K**) Rhodamine-phalloidin staining for actin (red), (**L**) anti-tubulin staining for microtubules (green), (**M**) DAPI staining (blue), and (**N**) merged images. Enlarged inset in (N) shows actin enrichment in growth cone. Scale bars: 5 µm. (**O**) DIC time-lapse series of cellular activities in the dendrite-like extension process of a neuron isolated from L4 stage worms. Dotted lines represent baseline positions of each of three intracellular motilities. Red arrowheads: appearance and disappearance of a large protrusion. Yellow arrowheads: a relative static protrusion. Blue arrowheads: rapid forward movement of a protrusion. Interval between each frame is 5 sec. Scale bar: 1 µm.

**Figure 5:**
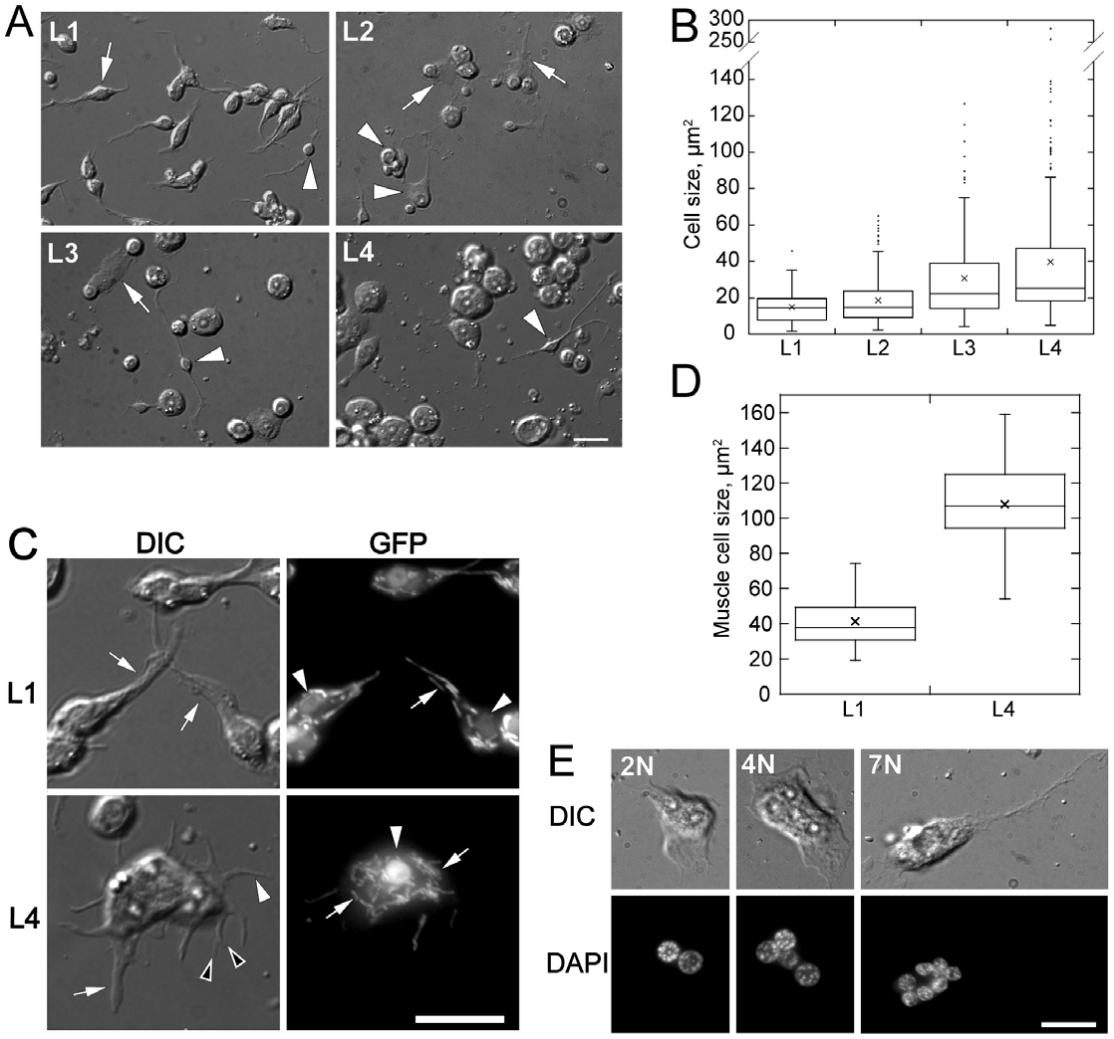
Cell sizes increase and cell morphologies vary with larval stage. (**A**) DIC micrographs of cells from L1–L4 worms one day after isolation (*stage indicated in each panel*). The fraction of large cells increases with progressive larval stages. Neurons (*arrowheads*) are found in cultures from all stages. Muscle is the major cell type in L1 isolates (*arrow*). Large cells from L2–L4 isolates are round with large flat protrusions (*arrows*). (**B**) Box and whisker plot of distribution of cell area of L1 (n = 205), L2 (n = 209), L3 (n = 176) and L4 (n = 238) cells one day after isolation. Central lines and boxes represent median and upper and lower quartiles of each distribution. Whiskers represent the robust range (quartiles±1.5x the interquartile distance). Means and outliers are show as crosses and dots. (**C**) DIC and fluorescence micrographs of L1 and L4 stage GFP positive cells isolated from *myo-3*::GFP strain, which expresses GFP in the nuclei and mitochrondria of muscle cells. DIC: L1 muscle cells have either one (*arrows*) or two (not shown) cell processes. L4 muscle cells show multiple cell processes, including wide (*arrow*), thin (*white arrowhead*) and bifurcated (*black arrowheads*) processes. GFP: Cells isolated from L1 and L4 show GFP expression in both nucleus (*arrowhead*) and mitochondria (*arrows*). (**D**) Box and whisker plot of distribution of cell area of L1 and L4 muscle cells (L1: n = 23; L4: n = 19). (**E**) DIC and fluorescence micrographs of fixed multinucleate (DAPI) cells from L4 isolates. Examples of 2, 4, and 7 nuclei (left to right) per cell are shown. All scale bars are 10 µm.

We next examined microtubule and actin localization in GFP-positive neurons isolated from the *unc-119*::GFP strain. In *C. elegans*, neurons are born mainly during embryogenesis and neural generation is completed by the L2 stage [46]. However, many neurons undergo post-mitotic development during which the neural cell bodies, clustered in ganglia at the head and tail, generate long, thin dendrite- and axon-like processes. These processes show local swellings of vesicle clusters that form at synaptic regions. Dendrite- and axon-like processes and neurite branches are characterized by microtubule bundles. Actively migrating axons terminate in a growth cone consisting of a dense, peripheral actin network that excludes all but a few microtubules bundles [47], [48]. In L1 derived GFP-positive cells from the *unc-119*::GFP strain, we found that microtubules were present in both cell bodies and neuronal processes (Figure **4**C, H and L), but that microtubules were excluded from the periphery of protrusions, where actin was highly expressed (Figure **4**K–N), a cytoskeletal arrangement that is typical of the leading edges of motile cells. Cultured neurons varied in the number and size of projections, and some isolated GFP-positive cells developed wide protrusions with strong microtubule staining (Figure **4**E, J and N). Actin-rich protusions, which are reminiscent of motile cell lamellipodia, appeared at one or more points along the length of each neuronal process, and showed dynamic protrusive activity (Figure **4**O). These actin rich structures were likely active growth cones of neuronal processes or active lamellipodia of neurons capable of motility. Although neuronal processes were not present in freshly isolated cells, we observed that unlabeled cultured neurons from all larval stages were capable of forming long dendritic and axonal processes (Figure **5**A, arrowheads). Because most neurons begin to form processes by stage L2, it is likely that isolated neurons can regenerate processes lost during isolation.

### Isolated larval cells exhibit developmental stage specific morphologies and behavior

Nematodes actively regulate cell and organ size during postembryonic development [49]. L4 larvae are about 2-fold thicker and 2.4-fold longer than L1 despite the fact that somatic cells only increase 1.7-fold in number [18]. Thus, based on stage specific estimates of nematode volume [50] and number of nuclei [18], we estimate that average cell size increases roughly two-fold from L1 to L4. We measured cell body area in cells isolated from different larval stages and found that cells isolated from L4 stage larvae were on average 2.7-fold larger than L1 derived cells (Figure **5**B). Cells from different larval stages also adopted distinct morphologies. For example, while the majority of L1 derived cells were spindle-shaped with single or double processes, many later stage cells had round cell bodies and extended wide cytoplasmic protrusions (Figure **5**A). We further examined these developmental stage-specific phenotypes using L1 and L4 derived cells from the *myo-3*:: GFP strain that expresses GFP in the nucleus and mitochondria of body wall muscle cells. Similar to the overall increase in cell size, we found that L4 GFP-positive muscle cells were 2.6 times larger than L1 GFP-positive muscle cells (Figure **5**D). Furthermore, isolated L4 muscle cells developed more cellular processes than L1 derived muscle cells. Observations from 3 independent cell isolations showed that L1 muscle cells developed from 1 to 2 wide processes with an average of 1.48±0.08 (n = 301) processes per cell, while L4 muscle cells developed an average of 8.4±1.0 (n = 45) processes of various thicknesses per cell (Figure **5**C). The developmental age of muscle cells *in vitro* correlated with muscle arm development. *In vivo*, L1 larvae muscle cells typically extend a maximum of 2 muscle arms, while older larval and adult muscle cells extend 3 to 5 muscle arms [51]. Therefore, our observations that L4 derived cells had 5.7-fold more muscle arms than L1 derived cells are consistent with the *in vivo* temporal pattern of muscle differentiation [51]. We note that *in vitro* cultured muscle cells have an abnormally large number of muscle arms (4 to 14) compared to *in vivo* muscle cells of comparable developmental age (3 to 5) [51]. Dixon and colleagues [51] have proposed that muscle arms extend passively as body wall muscles move away from the nerve cord during embryogenesis but switch to active extension during larval development. Because we observed active protrusion and retraction of muscle processes in culture (not shown), and larval muscle arm extension is highly regulated *in vivo*, it is possible that *in vitro* culture conditions induce ectopic muscle arm extension in L4 muscle cells due to the lack of late stage suppression that is normally found *in vivo*.

In addition to developmental stage specific muscle cell morphology, we observed large multinucleate cells in L4 cell culture, but rarely in cells isolated from younger nematodes (Figure **5**E). Approximately one third of somatic nuclei in the adult are found in syncytia [52]. For example, the Hyp7 epithelial syncytium, the largest somatic syncytium in *C. elegans* consists of 133 adult nuclei and forms a contiguous epidermal tubes that encircles the entire nematode body except for the extreme head and tail regions [52], [53], [54]. However, the multinucleate cells isolated from late larval stage worms are unlikely to be derived from the large Hyp7 syncytium, which would be broken apart on dissolution of the cuticle and mechanical disruption of cell contacts. The observed multinucleated cells may instead be derived from vulval, uterine and epithelial cell lineages that form syncytia of 4 to 16 nuclei during late larval development [55], [56]. During L4 stage, the vulval cells form tetra-nucleate and bi-nucleate syncytia that constitute the epithelial toroids of the vulva, while the uterine toroid cells form similar epithelial syncytia at the uterine lobes [55]. At the end of larval development, 16 seam cells terminally differentiate by fusing along their lateral axes to form the seam syncytium, which extends along the body length of the worm, and from which the adult alae structures are secreted [56]. The multinucleate cells observed in late larval stage cell isolations may thus be vulval, uterine, or seam cell epithelial in origin. Alternatively, developmentally older cells may have greater competence for cell fusion, an event that is normally restricted *in vivo* by active cellular mechanisms that are lost or absent in culture conditions, thus allowing older cells to form multinucleate cells *in vitro*. The rare L1 derived multinucleate cells may derive from a number of cell lineages that form syncytia during late embryogenesis and early larval development. For example, pharyngeal muscle cells form hexa-nucleate and bi-nucleate syncytia, and arcade cells of the anterior hypodermis form two epithelial syncytia that are part of the buccal cavity [57].

## Discussion

The investigation of cellular and subcellular processes in cultured cells is a mainstay experimental approach for the study of invertebrate and vertebrate model organisms. However, large scale cell culture in *C. elegans* has been limited to embryonically derived cells [5], [6]. Primary cultures of *C. elegans* embryonic cells terminally differentiate within 24 hours of isolation to resemble L1 stage cells [6], and thus post-embryonic cellular phenomena cannot be studied using these cells. We have developed a technically simple and efficient method for large-scale isolation and primary culture of cells from *C. elegans* larvae. Our method involves treatment of *C. elegans* larvae with a combination of detergent and reducing agent followed by protease digestion that effectively solubilizes the larval cuticle but does not kill cells. Large quantities of viable larval cells from synchronized L1 to L4 stage worms can be obtained using this method, and we have successfully isolated both high and low abundance larval cell types. Like embryonic cells that can be cultured for up to two weeks [5], [6], we have repeatedly cultured active larval cells for at least seven days. The isolated larval cells showed both cell type specific and developmental stage specific gene expression, morphologies and cell behaviors *in vitro*, indicating that isolated cells are normally differentiated and functional.

There are minor differences in the optimum attachment substrate for larval cells versus embryonic cells, but overall, the culture conditions, cell viability and cell yields of our larval cell isolation method are very similar to those of embryonic cell isolation as described by Strange and colleagues [5], [6]. Thus, *C. elegans* larval cells isolated according to our method should be amenable to the various cell biological techniques that have been used to study cultured embryonic cells, such as electrophysiology, RNAi, and fluorescence activated cell sorting (FACS) [5]. However, using isolated larval cells have several advantages over using embryonically derived cells. Embryonic isolation yields cells that are primarily from pre-comma stage embryos [5]. Since cultured *C. elegans* embryonic cells terminally differentiate after one day in isolation, it is possible that cell differentiation is not entirely normal compared to cells *in situ*. For example, transmembrane receptors and membrane channels characteristic of a cell type may not be expressed or properly localized in isolated embryonic cells [58]. In contrast to using isolated *C. elegans* embryonic cells, the ability to isolate and culture larval stage cells enables the investigator to specify cell isolation from a developmental stage at which the cell type of interest is properly differentiated. In addition, a greater variety of differentiated cell types are available from *C. elegans* larvae compared to embryos. Larval cells isolated by our method show developmental-stage specific morphologies for at least 72 hours, indicating that they at least partially maintain their original differentiation state and do not terminally differentiate within this period of time. Thus, larval cell culture can be used to investigate differences in cell physiology and behavior, cell autonomy, or track temporal changes in expression patterns, during larval development of a particular cell type.

It may be possible to manipulate culture conditions to maintain the undifferentiated or partial differentiation state of isolated larval cells. Cell plating density and the use of ‘feeder’ cells are two parameters that have substantial influence on differentiation and proliferative capability of cultured mammalian cells [59], [60], [61]. For example, mouse and human progenitor cells and induced pluripotent stem cells can be maintained in a self-renewal state by growth on a layer of ‘feeder’ fibroblast cells and similar techniques could be adapted for *C. elegans* larva cell culture [59], [62]. For *C. elegans* cell lineages that divide during larval development but do not terminally differentiate until the adult stage, such as the epidermal seam cells or vulval cells, isolation of larval cells and culture in conditions that prevent terminal differentiation would be the basis of establishing both primary and transformed cell lines, a cell biology tool that is currently not available for *C. elegans* research.

*C. elegans* larva cell culture has the normal disadvantages of any culture system, such as the absence of extracellular signaling due to the lack of cell to cell and cell to extracellular matrix contacts. Although we have shown that we can successfully isolate a low-abundance cell type that normally constitutes less that 1% of the *in vivo* cell population, our method may not be equally successful in isolating other low abundance cell types. However, in combination with embryonic cell culture, and a variety of existing experimental tools, including Western blots, subcellular localization, cell-specific profiling, RNAi, and FACS, our method to isolate and culture a variety of cell types from different post-embryonic developmental stages has the potential to substantially further the study of physiological, cellular, and molecular phenomena at the single cell and subcellular levels in *C. elegans*.

## Materials and Methods

### *C. elegans* strains and culture

The following *C. elegans* strains were used: N2 (wild type), DP132 [*unc-119::gfp*], PD4251 [*myo-3::Ngfp-lacZ*; *myo-3::Mtgfp*], NH3402 [*hlh8::gfp-caax*].

*Standard Culture:* For standard culture of *C. elegans* on solid medium, worms were maintained on NGM seeded with OP50 bacteria at either 15°C, 20°C, or 25°C.

### Axenic culture

For axenic liquid culture, *C. elegans* were first grown at 25°C at a density of 20,000 worms/plate on NEP plates seeded with NA22 bacteria [63]. Eggs were isolated from these worm cultures and grown in CeHR medium according to Szilagyi *et al.* and Nass and Hamza [34], [35] with minor modifications. 40,000–80,000 freshly hatched sterile L1 were seeded in 10 ml CeHR medium without antibiotics in a T-25 flask and grown at 22°C at 70 rpm in a shaker. After each generation, gravid adults were pelleted as above, eggs isolated and hatched in sterile M9 buffer, and L1 larvae were seeded into fresh, sterile CeHR media and grown to adulthood or the desired isolation stage. The first generation of nematodes grows slowly on CeHR (7–10 days), while successive generations grow at similar rates (4 days) to those on solid media [35]. Nematodes were allowed to adapt to CeHR media for at least one full generation before use.

### Larval cell isolation and culture

#### Worm synchronization

Eggs were released by lysing gravid adults with 0.5 M NaOH and 1.2% NaClO (bleach) for 5 min, pelleted by centrifugation at 1 min in a clinical centrifuge, and washed 3 times with sterile ddH_2_O. Eggs were hatched in sterile M9 and L1 were starved for 20–24 hrs at 20°C (22°C for CeHR culture).

### L1 cell isolation

Synchronized L1 were pelleted by centrifugation at 1 min in a clinical centrifuge, and M9 was removed by washing the pellet once with sterile ddH_2_O. Pelleted L1 were transferred to a 1.6 ml microfuge tubes and residual ddH_2_O was removed by centrifugation at 13,000 rpm for 2 min in a microcentrifuge. 20–40 µl L1 pellet was used for cell isolation. Worms were incubated in 200 µl freshly thawed sterile SDS-DTT solution (200 mM DTT, 0.25% SDS, 20 mM HEPES, pH 8.0, 3% sucrose, stored at −20°C) for 2 min at room temperature. Immediately after SDS-DTT treatment, 800 µl egg buffer (118 mM NaCl, 48 mM KCl, 2 mM CaCl_2_, 2 mM MgCl_2_, 25 mM HEPES, pH 7.3, osmolarity adjusted to 340 mOsm with sucrose) was added to the reaction. Worms were pelleted at 13,000 rpm for 1 min, and washed 5 times with 1 ml egg buffer. Pelleted SDS-DTT treated worms were then digested with 100 µl of 15 mg/ml pronase (Sigma-Aldrich, St. Louis, MO) in egg buffer at room temperature for 7 to 9 min. Mechanical disruption was applied during pronase digestion by pipetting the reaction up and down 60 times with a 1–200 µl tip pushing against the bottom of the 1.6 ml microfuge tube. The reaction was stopped by adding 900 µl L-15 medium (Invitrogen, Carlsbad, CA) supplemented with 10% fetal bovine serum (Invitrogen, Carlsbad, CA), 50 U/ml penicillin, and 50 µg/ml streptomycin (Sigma-Aldrich, St. Louis, MO) and adjusted to 340 mOsm [5]. Cells were pelleted by centrifugation at 10,000 rpm for 5 min at 4°C, and washed 2 times with L-15/FBS. The pellet was resuspended with 1 ml L-15/FBS and settled on ice for 30 min. The top 800 µl cell suspension devoid of large worm debris was transferred to a new tube and pelleted by centrifugation at 10,000 rpm for 5 min at 4°C.

### L2–L4 cell isolation

Worms grown in CeHR medium were harvested at approximately 26 hrs (L2), 34 hrs (L3) and 50 hrs (L4) post L1 seeding according to the growth rate reported by Szilagyi *et al.* [34]. Five ml CeHR culture was transferred each time from a T-25 flask into a 15 ml tube with 5 ml sterile ddH_2_O and mixed. Worms were pelleted at low speed for 5 seconds in a clinical centrifuge. The same procedure was applied to the other 5 ml of CeHR culture. Worms were washed 2–3 times with 10 ml ddH_2_O. In each wash, worms were pelleted for only 5 sec in a clinical centrifuge so that medium particles remained in the supernatant. The same procedure as L1 was followed subsequently for L2–L4 cell isolation, except that for L2–L4 cell isolation, worms were first treated with SDS-DTT for 4 min, and then digested in 15 mg/ml pronase for 20–25 min with pipetting for 140–160 strokes.

### Estimation of pelleted embryos or larvae

Eggs from gravid adults were prepared as above and either counted immediately or hatched overnight in sterile M9 and counted. To estimate the number of animals in suspension, we counted all animals from 5 µl of a 10 ml egg or L1 suspension on a dissecting microscope. Animals were concentrated in a clinical centrifuge, transferred to a 1.5 ml tube, and pelleted at 13,000 rpm for 1 min. The supernatant was removed, and centrifugation was repeated twice to remove any remaining supernatant. The pellet volume was marked on the tube and the pellet was discarded. Animals per µl of packed pellet were estimated from the starting number of animals in suspension and the final pellet volume estimated by refilling to the mark with ddH_2_O.

### Culture of isolated cells

Cell pellets were resuspended in fresh L-15/FBS. Suspended cells were counted in a haemocytometer, diluted to 5 to 6×10^6^ cells/ml and 30 to 40 µl of cell suspension was plated onto the center of a glass bottom dish (MatTek, Ashland, MA) or an acid-washed coverslip coated with 0.5 mg/ml peanut lectin (Sigma-Aldrich, St. Louis, MO). Cells were allowed to adhere overnight in a 20°C incubator without CO_2_ in Snapware plastic containers with a gas exchange hole and humidified with moist kimwipes. Unbound cells and worm debris were washed off with L-15 the next day and 2 ml fresh L-15/FBS was added to the dish.

### Cell viability assay

Cells grown on peanut lectin-coated glass bottom dishes were washed five times with egg buffer. Cells were incubated in 150 µl egg buffer containing 1 µM calcein-AM (Biotium, Hayward, CA) and 0.1 µM ethidium homodimer (Biotium, Hayward, CA) for 30 min at room temperature, and live and dead cells were quantified by fluorescence microscopy. Control cells were killed with 50% methanol in egg buffer and stained as above. No staining or very weak-green staining and bright red nuclear staining were observed in control experiments.

### Immunofluorescence

#### Microtubule and myosin staining

Cells were fixed with 1% paraformaldehyde in cytoskeleton buffer (10 mM MES, pH 6.1, 150 mM NaCl, 5 mM EGTA, 5 mM MgCl_2_, and 5 mM glucose) at room temperature for 30 min and then cold methanol at −20°C for 2 min, blocked with 5% BSA in PBS for 30 min, and incubated with mouse anti-tubulin primary antibody (Thermo Fisher Scientific, Fremont, CA; clone DM1A) or mouse anti-MHC-A primary antibody (Developmental Studies Hybridoma Bank, Iowa City, IA; clone 5–6). Cells were then stained with DyLight594-conjugated goat anti-mouse secondary antibody (Jackson ImmunoResearch Laboratories, West Grove, PA) and DAPI [5], [64].

### Actin and microtubule staining

Cells were fixed with 0.25% glutaraldehyde in cytoskeleton buffer for 15 min and permeabilized with 0.1% Triton X-100 in cytoskeleton buffer for 10 min. Autofluorescence was quenched 3 times with fresh 1 mg/ml sodium borohydride in cytoskeleton buffer. Cells were blocked with 5% BSA in PBS and stained with TRITC-phalloidin (Sigma-Aldrich, St. Louis, MO) for 20 min, incubated with mouse anti-tubulin primary antibody, and then stained with FITC goat anti-mouse secondary antibody (Jackson ImmunoResearch Laboratories, West Grove, PA) and DAPI [64].

### Microscopy

DIC and epifluorescence images were taken using Olympus IX81 inverted microscope (Olympus, Center Valley, PA). Images were acquired with 60× objective (UPlanSApo, NA = 1.40, Olympus) or 100× objective (Apo N, NA = 1.49, Olympus). Phase contrast images were taken with Olympus CKX41 tissue culture microscope (Olympus, Center Valley, PA) equipped with a 20× objective (LCAch N, NA = 0.40, Olympus).

## References

[1] Cowan AE, McIntosh JR (1985). Mapping the distribution of differentiation potential for intestine, muscle, and hypodermis during early development in Caenorhabditis elegans.. Cell.

[2] Edgar LG, McGhee JD (1988). DNA synthesis and the control of embryonic gene expression in C. elegans.. Cell.

[3] Goldstein B (1992). Induction of gut in Caenorhabditis elegans embryos.. Nature.

[4] Bloom L (1993). Genetic and molecular analysis of genes required for axon outgrowth in caenorhabditis elegans..

[5] Christensen M, Estevez A, Yin X, Fox R, Morrison R (2002). A primary culture system for functional analysis of *C. elegans* neurons and muscle cells.. Neuron.

[6] Strange K, Christensen M, Morrison R (2007). Primary culture of Caenorhabditis elegans developing embryo cells for electrophysiological, cell biological and molecular studies.. Nat Protoc.

[7] Zhang Y, Ma C, Delohery T, Nasipak B, Foat BC (2002). Identification of genes expressed in C. elegans touch receptor neurons.. Nature.

[8] Francis MM, Maricq AV (2006). Electrophysiological analysis of neuronal and muscle function in C. elegans.. Methods Mol Biol.

[9] Von Stetina SE, Watson JD, Fox RM, Olszewski KL, Spencer WC (2007). Cell-specific microarray profiling experiments reveal a comprehensive picture of gene expression in the C. elegans nervous system.. Genome Biol.

[10] Fox RM, Von Stetina SE, Barlow SJ, Shaffer C, Olszewski KL (2005). A gene expression fingerprint of C. elegans embryonic motor neurons.. BMC genomics.

[11] Fox RM, Watson JD, Von Stetina SE, McDermott J, Brodigan TM (2007). The embryonic muscle transcriptome of Caenorhabditis elegans.. Genome Biol.

[12] Meissner B, Warner A, Wong K, Dube N, Lorch A (2009). An integrated strategy to study muscle development and myofilament structure in Caenorhabditis elegans.. PLoS Genet.

[13] Caldwell KA, Tucci ML, Armagost J, Hodges TW, Chen J (2009). Investigating bacterial sources of toxicity as an environmental contributor to dopaminergic neurodegeneration.. PLoS One.

[14] Sobkowiak R, Lesicki A (2009). Genotoxicity of nicotine in cell culture of Caenorhabditis elegans evaluated by the comet assay.. Drug Chem Toxicol.

[15] Shih JD, Fitzgerald MC, Sutherlin M, Hunter CP (2009). The SID-1 double-stranded RNA transporter is not selective for dsRNA length.. RNA.

[16] Wilkins C, Dishongh R, Moore SC, Whitt MA, Chow M (2005). RNA interference is an antiviral defence mechanism in Caenorhabditis elegans.. Nature.

[17] Zhou KM, Dong YM, Ge Q, Zhu D, Zhou W (2007). PKA activation bypasses the requirement for UNC-31 in the docking of dense core vesicles from C. elegans neurons.. Neuron.

[18] Hirsh D, Oppenheim D, Klass M (1976). Development of the reproductive system of *Caenorhabditis elegans*.. Dev Biol.

[19] Sulston JE, Horvitz HR (1977). Post-embryonic cell lineages of the nematode, *Caenorhabditis elegans*.. Dev Biol.

[20] Strange K (2003). From genes to integrative physiology: ion channel and transporter biology in Caenorhabditis elegans.. Physiol Rev.

[21] Brockie PJ, Mellem JE, Hills T, Madsen DM, Maricq AV (2001). The *C. elegans* glutamate receptor subunit NMR-1 is required for slow NMDA-activated currents that regulate reversal frequency during locomotion.. Neuron.

[22] Nickell WT, Pun RY, Bargmann CI, Kleene SJ (2002). Single ionic channels of two *Caenorhabditis elegans* chemosensory neurons in native membrane.. J Membr Biol.

[23] Richmond JE, Jorgensen EM (1999). One GABA and two acetylcholine receptors function at the *C. elegans* neuromuscular junction.. Nat Neurosci.

[24] Teramoto T, Iwasaki K (2006). Intestinal calcium waves coordinate a behavioral motor program in *C. elegans*.. Cell Calcium.

[25] Roy PJ, Stuart JM, Lund J, Kim SK (2002). Chromosomal clustering of muscle-expressed genes in Caenorhabditis elegans.. Nature.

[26] Von Stetina SE, Watson JD, Fox RM, Olszewski KL, Spencer WC (2007). Cell-specific microarray profiling experiments reveal a comprehensive picture of gene expression in the C. elegans nervous system.. Genome Biol.

[27] Yang Z, Edenberg HJ, Davis RL (2005). Isolation of mRNA from specific tissues of Drosophila by mRNA tagging.. Nucleic Acids Res.

[28] Page AP, Johnstone IL (2007). The cuticle.. WormBook.

[29] Cox GN, Kusch M, Edgar RS (1981). Cuticle of *Caenorhabditis elegans*: its isolation and partial characterization.. J Cell Biol.

[30] Austin J, Kenyon C (1994). Cell contact regulates neuroblast formation in the *Caenorhabditis elegans* lateral epidermis.. Development.

[31] Johnstone IL (1994). The cuticle of the nematode *Caenorhabditis elegans*: a complex collagen structure.. Bioessays.

[32] Cox GN, Staprans S, Edgar RS (1981). The cuticle of *Caenorhabditis elegans*. II. Stage-specific changes in ultrastructure and protein composition during postembryonic development.. Dev Biol.

[33] Papadopoulos NG, Dedoussis GV, Spanakos G, Gritzapis AD, Baxevanis CN (1994). An improved fluorescence assay for the determination of lymphocyte-mediated cytotoxicity using flow cytometry.. J Immunol Methods.

[34] Szilagyi M, Gehman E, Lapenotiere H, Lewis J, Clegg E (2006). Global Alterations in Gene Expression During Organophosphate Pesticide Intoxication and Recovery: Interim Report. Defense Technical Information Center Website.. http://handle.dtic.mil/100.2/ADA469210.

[35] Nass R, Hamza I (2007). The Nematode *C. elegans* as an Animal Model to Explore Toxicology *In Vivo*: Solid and Axenic Growth Culture Conditions and Compound Exposure Parameters.. Curr Protocols in Toxicology.

[36] Honda S, Epstein HF (1990). Modulation of muscle gene expression in Caenorhabditis elegans: differential levels of transcripts, mRNAs, and polypeptides for thick filament proteins during nematode development.. Proc Natl Acad Sci U S A.

[37] Fire A, Xu S, Montgomery MK, Kostas SA, Driver SE (1998). Potent and specific genetic interference by double-stranded RNA in *Caenorhabditis elegans*.. Nature.

[38] Maduro M, Pilgrim D (1995). Identification and cloning of unc-119, a gene expressed in the Caenorhabditis elegans nervous system.. Genetics.

[39] Harfe BD, Vaz Gomes A, Kenyon C, Liu J, Krause M (1998). Analysis of a Caenorhabditis elegans Twist homolog identifies conserved and divergent aspects of mesodermal patterning.. Genes Dev.

[40] Schachat F, Garcea RL, Epstein HF (1978). Myosins exist as homodimers of heavy chains: demonstration with specific antibody purified by nematode mutant myosin affinity chromatography.. Cell.

[41] Okkema PG, Harrison SW, Plunger V, Aryana A, Fire A (1993). Sequence requirements for myosin gene expression and regulation in Caenorhabditis elegans.. Genetics.

[42] Ardizzi JP, Epstein HF (1987). Immunochemical localization of myosin heavy chain isoforms and paramyosin in developmentally and structurally diverse muscle cell types of the nematode Caenorhabditis elegans.. J Cell Biol.

[43] Miller DM, Stockdale FE, Karn J (1986). Immunological identification of the genes encoding the four myosin heavy chain isoforms of Caenorhabditis elegans.. Proceedings of the National Academy of Sciences of the United States of America.

[44] Miller DM, Ortiz I, Berliner GC, Epstein HF (1983). Differential localization of two myosins within nematode thick filaments.. Cell.

[45] Altun ZF, Hall DH (2009). Muscle system, somatic muscle.. WormAtlas.

[46] Altun ZF, Hall DH (2009). Nervous system, general description.. WormAtlas.

[47] Durbin RM (1987). Studies on the Development and Organisation of the Nervous System of Caenorhabditis elegans..

[48] Lowery LA, Van Vactor D (2009). The trip of the tip: understanding the growth cone machinery.. Nat Rev Mol Cell Biol.

[49] Wang J, Tokarz R, Savage-Dunn C (2002). The expression of TGFbeta signal transducers in the hypodermis regulates body size in *C. elegans*.. Development.

[50] Altun ZF, Hall DH, Altun ZF, Herndon LA, Crocker C, Lints R, Hall DH (2009). Introduction.. WormAtlas.

[51] Dixon SJ, Roy PJ (2005). Muscle arm development in *Caenorhabditis elegans*.. Development.

[52] Hedgecock EM, White JG (1985). Polyploid tissues in the nematode *Caenorhabditis elegans*.. Dev Biol.

[53] Sulston JE, Schierenberg E, White JG, Thomson JN (1983). The embryonic cell lineage of the nematode Caenorhabditis elegans.. Developmental biology.

[54] Shemer G, Podbilewicz B (2000). Fusomorphogenesis: cell fusion in organ formation.. Developmental dynamics : an official publication of the American Association of Anatomists.

[55] Lints R, Hall DH (2009). Reproductive system, egglaying apparatus.. WormAtlas.

[56] Altun ZF, Hall DH (2009). Epithelial system, seam cells.. WormAtlas.

[57] Podbilewicz B (2006). Cell fusion.. WormBook.

[58] Bianchi L, Driscoll M (2006). Culture of embryonic C. elegans cells for electrophysiological and pharmacological analyses.. WormBook.

[59] Lin S, Talbot P (2011). Methods for culturing mouse and human embryonic stem cells.. Methods Mol Biol.

[60] Moldaver MV, Yegorov YE (2009). Sparse plating increases the heterogeneity of proliferative potential of fibroblasts.. Mechanisms of Ageing and Development.

[61] Portela VM, Zamberlam G, Price CA (2010). Cell plating density alters the ratio of estrogenic to progestagenic enzyme gene expression in cultured granulosa cells.. Fertil Steril.

[62] Pan C, Hicks A, Guan X, Chen H, Bishop CE (2010). SNL fibroblast feeder layers support derivation and maintenance of human induced pluripotent stem cells.. J Genet Genomics.

[63] Merritt C, Seydoux G (2010). Transgenic solutions for the germline.. WormBook.

[64] Prast J, Gimona M, Small JV, Celis JE (2006). Immunofluorescence microscopy of the cytoskeleton: combination with green fluorescent protein tags.. Cell Biology: A Laboratory Handbook (3rd ed).

